# A Survey of Infection in Allogenic Hematopoietic Stem Cell Transplantation in Patients with Acute Myeloid Leukemia

**Published:** 2018-08-01

**Authors:** S. R. Safayi, f. Shahi, M. Ghalamkari, M. Mirzania, M. Khatuni, F. Hirmandi Niasar

**Affiliations:** 1 Assisstant Professor of Hematology Oncology, Tehran University of Medical Sciences, Tehran, Iran; 2 Associated Professor of Hematology Oncology, Tehran University of Medical Sciences, Tehran, Iran; 3 Fellowship of Hematology Oncology, Tehran University of Medical Sciences, Tehran, Iran; 4 Internal Medicine Specialist Tehran University of Medical Sciences; 5 Researcher at Tehran University of Medical Sciences, Tehran, Iran

**Keywords:** Bone marrow transplantation; Infection; Acute myeloid leukemia

## Abstract

**Background:**

Allogeneic hematopoietic stem cell transplantation (HSCT) is a potentially cure for acute myeloid leukemia (AML). Patients who undergone HSCT are at increased risk of infection due to impaired immunity.

**Objective:**

To evaluate the rate of bacterial, viral and fungal infection and its relationship with 2-year overall survival of AML patients who had undergone HSCT.

**Methods:**

This was a retrospective cross-sectional study of 49 patients who underwent allogenic bone marrow transplantation (BMT) from full-matched donors at BMT Center, Imam Khomeini Hospital Complex, Tehran, Iran, from 2006 to 2013. All autologous transplantations and promyelocytic leukemia (PML) transplantations were excluded.

**Results:**

All patients, except for one, had fever for a mean of 7 days post-transplantation and received broad-spectrum antibiotic. The rate of severe sepsis was 6.1%. None of the patients developed fungal infection during admission. The rate of admission due to sepsis after discharge was 27% in the alive group (mean onset of 54 days), and 73% in the deceased group (mean onset of 52 days) (p<0.05). The most common site of infection was lung (70%). The rate of cytomegalovirus (CMV) antigenemia (positive PP65) was 20% during the 2-year period after HSCT.

**Conclusion:**

The rate of infection was a negative prognostic factor for 2-year overall survival. The rate of CMV antigenemia is less than similar studies (51%), which could be due to full-matched donor-recipients requiring less immunosuppression.

## INTRODUCTION

Autologous or allogenic hematopoietic stem cell transplantation is currently used as a choice of treatment for patients with acute myeloid leukemia (AML), leading to long-term remission and cure [1-3]. Patients undergoing hematopoietic stem cell transplantation are at increased risk for bacterial and fungal infection due to invasive conditioning regimen, central venous line placement and prolonged immune suppression [4]. Infection is an important cause of morbidity and mortality after bone marrow transplantation (BMT) (*e.g.*, up to 15% mortality rate) [5]. Therefore, early identification of high risk patients and applying more effective preventive and supportive strategies for reducing the rates of infection may potentially improve the survival rates [5-8]. Sepsis due to neutropenia is common during the first 30 days after BMT. Furthermore, factors related to acute graft versus host disease (GVHD) or its treatment may cause sepsis in the 30–100 days after transplantation [4].

There is a concern that higher rates of infection in developing countries may be associated with higher incidence of morbidity and mortality [4]. The present study aimed to analyze the pattern of post-transplantation infection in patients who underwent allogenic BMT at Imam Khomeini BMT Center during an 8-year period.

## MATERIALS AND METHODS

This was a cross-sectional study of 49 patients diagnosed with AML. The study was performed at the BMT Center of Imam Khomeini Hospital Complex, Tehran, Iran. The inclusion criteria were all patients who had been diagnosed with AML and had received allo-hematopoietic stem cell transplantation (HSCT) from 2006 to 2013. The exclusion criteria were all patients with other malignancies and/or promyelocytic leukemia (PML) and those who had received autologous stem cell transplantation. All patients received allo-stem cells from fully HLA-matched donors.

The preparative regimen for all patients was cyclophosphamide 5 mg/kg/day on day 1 and 2 and busulphan 4 mg/kg/day on days 3 to 6.

Antimicrobial prophylaxis regimen was acyclovir 15 mg/kg/day, sulfamethoxazole/trimethoprim once daily and itraconazole 200 mg/day, which were all started on day +2, and continued for six months. The patient was considered to have fever if the measured core body temperature was above 38.5 °C once or above 38.3 °C twice.

Sepsis was defined according to the 2001 revised criteria, consisting of systemic inflammatory response syndrome (SIRS) plus some degrees of organ dysfunction (in severe sepsis) [9]. Diagnosis of cytomegalovirus (CMV) antigenemia was based on the PCR technique. The site of infection was determined by clinical evaluation, radiography (*e.g.*, chest x-ray), or positive culture from blood, urine, sputum, abscess, or catheter samples. Broad-spectrum antibiotics were started when fever was detected or infection was documented.

**Statistical Analysis**

SPSS^®^ ver 22.0 for Windows^®^ (SPSS Inc, Chicago, IL, USA) was used for data analysis. Results are presented as mean±SD for quantitative variables and as absolute frequencies and percentages for categorical variables. Normal distribution of all data was checked using the Kolmogorov-Smirnoff test. Categorical variables were analyzed using χ^2^ test or Fisher’s exact test when more than 20% of cells with expected count of less than 5 were observed. Quantitative variables were analyzed using *Student’s t* test or Mann-Whitney U test. Survival rate was analyzed using the Kaplan-Meier curves and the log-rank test. A p value <0.05 was considered statistically significant.

## RESULTS

A total of 49 patients with AML were registered to have allogeneic-HSCT during 2006-2013 at the BMT Center of Imam Khomeini Hospital Complex. Baseline characteristics of the study sample are summarized in Table 1.

**Table 1:** Baseline characteristics of the study sample

Variable	n (%)
Age (yrs)	
≤40	36 (74)
>40	13 (27)
Sex	
Female	27 (55)
Male	22 (45)
Recipient-donor sex	
Matched	18 (37)
Unmatched	31 (63)
Blood group	
Matched	28 (58)
Unmatched	20 (42)
Donor type	
Sibling	48 (98)
Father	1 (2)
Underlying disease	
Lymphoma	3 (6)
CML	2 (4)
Autoimmune hemolytic anemia	1 (2)
Diabetes	4 (8)
Tuberculosis	1 (2)

**Figure 1:**
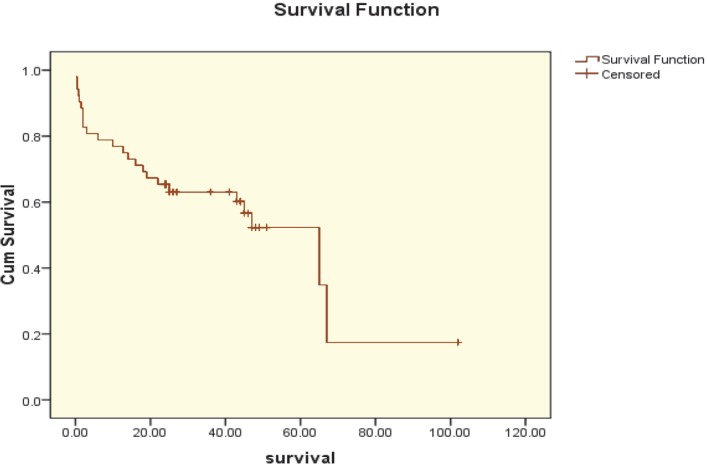
The Kaplan-Mayer survival curve in AML patients treated with allogeneic stem cell transplantation

The overall two-year survival rate of patients who underwent transplantation was 65.4%±6.6% (Fig 1). As assessed by Cox proportional hazard analysis, the main predictor for reduced 2-year survival after transplantation was post-transplantation infection (p=0.021).

All patients, but one, had fever for a mean of seven days after transplantation and received broad-spectrum antibiotics. The rate of severe sepsis during the admission for transplantation was 6.1%. No fungal infection was detected. None of the patients had viral hepatitis (Table 2).

**Table 2:** Complications during the admission for transplantation

Complication	n (%)
Fever	48 (98)
Severe sepsis	3 (6)
Fungal infection	0 (0)
Viral hepatitis	0 (0)

The rate of admission after discharge due to sepsis was 27% in the alive group (mean onset of 54 days) and 73% in the deceased group (mean onset of 52 days) (p<0.05, Table 3). The most common site of infection was lung (47%) (Table 4). The rate of CMV antigenemia after transplantation (positive PP65) was 20% during a 2-year period after HSCT.

**Table 3:** The rate of sepsis after transplantation

Group	Post-BMT sepsis, n (%)	Post-BMT sepsis onset (days), Mean±SD
Alive (two years post-BMT)	4 (27)	54.1±18
Deceased (two years post-BMT)	11 (73)	52.3±20
Total	15 (100)	52.9±20

**Table 4:** Sites of post-BMT infection

Site of post-BMT infection	n (%)
Lung	7 (47)
Skin	2 (13)
Central nervous system	1 (6)
Unknown origin	5 (34)
Total	15 (100)

## DISCUSSION

The present study was an overview of post-BMT infection rate in patients with AML. The 2-year overall survival was 65.4%±6%. Other studies have reported different 2-year overall survival according to the patients’ characteristics and comorbid diseases. In a study by Hamidieh, *et. al.*, on HSCT in Iranian children, the overall survival during two years after allogenic transplantation in leukemic patients was 60%–70% [10]. Similar results were reported in another study from an Italian center [11].

Infection is one of the most common causes of mortality in patients who undergo transplantation [12]. In the study conducted by Hamidieh, *et. al.*, infections accounted for 18.5% of all deaths [10]. In our study, 11 patients in the deceased group got infection (Table 3), which accounted for 22% of all deaths.

Fever and neutropenia after transplantation are common and require broad-spectrum antibiotics. Almost all of our patients (98%) had fever by a mean onset of seven days after transplantation and received empiric antibiotic therapy. This rate of fever is higher than similar BMT centers; an Indian study reports fever in 82.5% of patients post-HSCT [4], while in western studies, this rate varies between 60% and 90% [6]. In a study conducted by Luznik, *et. al.*, the rate of fever with neutropenia was 51% during the first 60 days after transplantation, and infections without neutropenia accounted for 22% of post-BMT admissions. The Luznik article does not mention the mean day of fever onset [13]. Hayatshahi and colleagues, report a 63.2% rate of neutropenic fever in adult BMT Department of Shariati Hospital, Tehran, Iran, consisting of both autologous and allogenic transplantations with different underlying diseases [14]. In our study, neutropenic *vs*. non-neutropenic fever was not specified. 

One of the limitations of our study was lack of enough data to report the frequency of bacterial species in febrile and septic patients. We did not detect any fungal infection during admission. However, the incidence of fungal infection varies from 4% to 30% in different transplant centers [15, 16]; studies from Asian countries like India and Israel have typically observed higher rates (19%) [4, 17].

One of the patients had pulmonary tuberculosis before transplantation, which was completely cured and did not reactivate. Surprisingly, none of the transplant recipients in our study was infected by tuberculosis. The incidence rate of the disease, however, varies between 1% and 12% in developing countries [18, 19].

Viral pathogens including herpes simplex and herpes zoster were not detected in this study. The worldwide incidence of viral infections, however, is about 8%–10% in similar studies [20, 21]. The incidence of CMV infection was not evaluated in our study, but the rate of CMV antigenemia during the 2-year period after transplantation was 20.4%, which was lesser than that reported in similar studies (51%) [22]. This difference could be due to full-match donor-recipient profile that requires less immunosuppressive therapy.

In conclusion, we found that the overall 2-year survival rate was similar to other BMT centers, but the rate of fever after transplantation was higher, and the incidence of viral, fungal, and tuberculosis infections was lesser than that reported in other centers.
